# Pulmonary Hypertension in Adults with Congenital Heart Disease: Real-World Data from the International COMPERA-CHD Registry

**DOI:** 10.3390/jcm9051456

**Published:** 2020-05-13

**Authors:** Harald Kaemmerer, Matthias Gorenflo, Dörte Huscher, David Pittrow, Christian Apitz, Helmut Baumgartner, Felix Berger, Leonhard Bruch, Eva Brunnemer, Werner Budts, Martin Claussen, Gerry Coghlan, Ingo Dähnert, Michele D’Alto, Marion Delcroix, Oliver Distler, Sven Dittrich, Daniel Dumitrescu, Ralf Ewert, Martin Faehling, Ingo Germund, Hossein Ardeschir Ghofrani, Christian Grohé, Karsten Grossekreymborg, Michael Halank, Georg Hansmann, Dominik Harzheim, Attila Nemes, Kalman Havasi, Matthias Held, Marius M. Hoeper, Michael Hofbeck, Wolfgang Hohenfrost-Schmidt, Elena Jurevičienė, Lina Gumbienè, Hans-Joachim Kabitz, Hans Klose, Thomas Köhler, Stavros Konstantinides, Martin Köestenberger, Rainer Kozlik-Feldmann, Hans-Heiner Kramer, Cornelia Kropf-Sanchen, Astrid Lammers, Tobias Lange, Philipp Meyn, Oliver Miera, Katrin Milger-Kneidinger, Rhoia Neidenbach, Claus Neurohr, Christian Opitz, Christian Perings, Bjoern Andrew Remppis, Gabriele Riemekasten, Laura Scelsi, Werner Scholtz, Iveta Simkova, Dirk Skowasch, Andris Skride, Gerd Stähler, Brigitte Stiller, Iraklis Tsangaris, Carmine Dario Vizza, Anton Vonk Noordegraaf, Heinrike Wilkens, Hubert Wirtz, Gerhard-Paul Diller, Ekkehard Grünig, Stephan Rosenkranz

**Affiliations:** 1 Deutsches Herzzentrum München, Klinik für Angeborene Herzfehler und Kinderkardiologie, München, Technische Universität München, 80636 Munich, Germany; Neidenbach@dhm.mhn.de; 2 Universitätsklinikum Heidelberg, Zentrum für Kinder- und Jugendmedizin, Angelika-Lautenschläger-Klinik, 69120 Heidelberg, Germany; 3 Institute of Biometry and Clinical Epidemiology, and Berlin Institute of Health, Charité Universitätsmedizin, 10117 Berlin, Germany; doerte.huscher@charite.de; 4 Medical Faculty, Institute for Clinical Pharmacology, Technical University, 01307 Dresden, Germany; david.pittrow@mailbox.tu-dresden.de; 5 GWT-TUD GmbH, Pharmacoepidemiology, 01307 Dresden, Germany; 6 Universitätsklinik für Kinder- und Jugendmedizin, Sektion Pädiatrische Kardiologie, 89075 Ulm, Germany; christian.apitz@uniklinik-ulm.de; 7 Universitätsklinik Münster, Klinik für Angeborene (EMAH) und Erworbene Herzfehler, 48149 Münster, Germany; helmut.baumgartner@ukmuenster.de (H.B.); gerhard.diller@ukmuenster.de (G.-P.D.); 8 Deutsches Herzzentrum Berlin, Klinik für Angeborene Herzfehler/Kinderkardiologie, 13353 Berlin, Germany; felix.berger@charite.de (F.B.); miera@dhzb.de (O.M.); 9 Unfallkrankenhaus Berlin, Klinik für Innere Medizin, 12683 Berlin, Germany; leonhard.bruch@ukb.de; 10 Medizinische Universitätsklinik (Krehl-Klinik), Klinik für Kardiologie, Angiologie und Pneumologie (Innere Medizin III), 69120 Heidelberg, Germany; eva.brunnemer@med.uni-heidelberg.de; 11 UZ Leuven, Congenital and Structural Cardiology, Campus Gasthuisberg, 3000 Leuven, Belgium; werner.budts@uzleuven.be; 12 LungenClinic Grosshansdorf, Fachabteilung Pneumologie, 22927 Großhansdorf, Germany; m.claussen@kh-grosshansdorf.de; 13 Royal Free Hospital, Cardiology, London NW3 2QG, UK; gerry.coghlan@nhs.net; 14 Herzzentrum Leipzig GmbH, Klinik für Kinderkardiologie, 04289 Leipzig, Germany; ingo.daehnert@helios-gesundheit.de; 15 Ospedale Monaldi, 80131 Napoli, Italy; mic.dalto@tin.it; 16 Department of Respiratory Diseases, University Hospitals of Leuven, 3000 Leuven, Belgium; marion.delcroix@uzleuven.be; 17 Universitätsspital Zürich, Klinik für Rheumatologie, 8091 Zürich, Switzerland; Oliver.Distler@usz.ch; 18 Universitätsklinikum Erlangen, Kinderkardiologie, 91054 Erlangen, Germany; sven.dittrich@uk-erlangen.de; 19 HDZ NRW, Klinik für Thorax- und Kardiovaskularchirurgie, 32545 Bad Oeynhausen, Germany; ddumitrescu@hdz-nrw.de; 20 Universitätsmedizin Greifswald, Zentrum für Innere Medizin, Klinik und Poliklinik für Innere Medizin B, 17475 Greifswald, Germany; ewert@uni-greifswald.de; 21 Klinikum Esslingen GmbH, Klinik für Kardiologie, Angiologie und Pneumologie, 73730 Esslingen a.N., Germany; m.faehling@klinikum-esslingen.de; 22 Uniklinik Köln—Herzzentrum, Klinik und Poliklinik für Kinderkardiologie, 50937 Köln, Germany; ingo.germund@uk-koeln.de; 23 Universitätsklinikum Gießen und Marburg GmbH, Medizinische Klinik II/V, 35392 Gießen, Germany; Ardeschir.Ghofrani@innere.med.uni-giessen.de; 24 Evangelische Lungenklinik Berlin, Klinik für Pneumologie, 13125 Berlin, Germany; Christian.Grohe@jsd.de; 25 Kinderherzzentrum und Zentrum für Angeborene Herzfehler, Justus-Liebig Universität, Zentrum für Kinderheilkunde, Abteilung Kinderkardiologie, 35390 Giessen, Germany; karsten.g.kreymborg@paediat.med.uni-giessen.de; 26 Universitätsklinikum Carl Gustav Carus der Technischen Universität Dresden, Medizinische Klinik und Poliklinik I, 01307 Dresden, Germany; Michael.Halank@uniklinikum-dresden.de; 27 Medizinische Hochschule Hannover, Zentrum für Pulmonale Hypertonie im Kindesalter/Klinik für pädiatrische Kardiologie und Intensivmedizin, 30625 Hannover, Germany; hansmann.georg@mh-hannover.de; 28 Waldburg Zeil Kliniken Gmbh & Co. KG, Fachkliniken Wangen, Lungenzentrum Süd-West, Klinik für Pneumologie, Beatmungsmedizin und Allergologie, 88239 Wangen im Allgäu, Germany; dominik.harzheim@wz-kliniken.de (D.H.); philipp.meyn@wz-kliniken.de (P.M.); 29 2nd Dep. of Internal Medicine and Cardiology Center Hungary, Faculty of Medicine, Szent-Györgyi Albert Clinical Center, University of Szeged, 6725 Szeged, Hungary; attila.nemes.md@gmail.com (A.N.); havasi.kalman@gmail.com (K.H.); 30 Missionsärztliche Klinik gGmbH, Abteilung für Innere Medizin, 97074 Würzburg, Germany; matthias.held@missioklinik.de; 31 Medizinische Hochschule Hannover, Abt. Pneumologie, 30625 Hannover, Germany; Hoeper.Marius@mh-hannover.de; 32 Universitätsklinik für Kinder- und Jugendmedizin Tübingen, Kinderkardiologie, Pulmologie, Intensivmedizin, 72076 Tübingen, Germany; michael.hofbeck@med.uni-tuebingen.de; 33 Kardiologie–Angiologie–Pneumologie, II. Medizinische Klinik des Klinikums Coburg, 96450 Coburg, Germany; wolfgang.hohenforst-schmidt@klinikum-coburg.de; 34 Faculty of Medicine of Vilnius University; Referal Centre of Pulmonary Hypertension, Vilnius University Hospital Santaros klinikos, 08661 Vilnius, Lithuania; Elena.Jureviciene@santa.lt (E.J.); lina.gumbiene@santa.lt (L.G.); 35 Gemeinnützige Krankenhausbetriebsgesellschaft Konstanz mbH, Medizinische Klinik II, 78464 Konstanz, Germany; hans-joachim.kabitz@glkn.de; 36 Universitätsklinikum Hamburg Eppendorf, Studienzentrale Pneumologie, 20251 Hamburg, Germany; klose@uke.de; 37 Universitätsklinikum Freiburg, Medizinische Klinik, Abteilung Pneumologie, 79106 Freiburg, Germany; thomas.koehler@uniklinik-freiburg.de; 38 Universitätsmedizin Mainz, Center for Thrombosis and Hemostasis, 55131 Mainz, Germany; stavros.konstantinides@unimedizin-mainz.de; 39 LKH - Univ. Klinikum Graz, Universitätsklinik für Kinder- und Jugendheilkunde, Abteilung für Pädiatrische Kardiologie, 8036 Graz, Austria; martin.koestenberger@medunigraz.at; 40 Universitäres Herzzentrum Hamburg, Klinik und Poliklinik für Kinderkardiologie, 20251 Hamburg, Germany; r.kozlik-feldmann@uke.de; 41 Universitätsklinikum Schleswig-Holstein, Klinik für angeborene Herzfehler & Kinderkardiologie (Haus 9), 24105 Kiel, Germany; H2Kramer@gmail.com; 42 Universitätsklinikum Ulm, Klinik für Innere Medizin II/Pneumologie, 89081 Ulm, Germany; Cornelia.kropf@uniklinik-ulm.de; 43 Westfälische Wilhelms-Universität Münster, Klinik für Kinder- und Jugendmedizin - Pädiatrische Kardiologie, 48149 Münster, Germany; Astrid.Lammers@ukmuenster.de; 44 Universitätsklinikum Regensburg, Medizinische Klinik und Poliklinik II, 93053 Regensburg, Germany; tobias.lange@klinik.uni-regensburg.de; 45 Klinikum der Universität München, Medizinische Klinik und Poliklinik V, 80336 München, Germany; Katrin.Milger@med.uni-muenchen.de; 46 Klinik Schillerhöhe, Abteilung für Pneumologie und Beatmungsmedizin, 70839 Gerlingen, Germany; claus.neurohr@klinik-schillerhoehe.de; 47 DRK Kliniken Berlin Westend, Klinik für Innere Medizin, Schwerpunkt Kardiologie, 14050 Berlin, Germany; c.opitz@drk-kliniken-berlin.de; 48 Klinikum Lünen, St. Marien-Hospital GmbH, 44534 Lünen, Germany; Perings.Christian@klinikum-luenen.de; 49 Herz- und Gefäßzentrum Bad Bevensen, 29549 Bad Bevensen, Germany; b.remppis@hgz-bb.de; 50 Clinic of Rheumatology and Clinical Immunology, University of Lübeck, University Clinic Schleswig Holstein, 23562 Lübeck, Germany; Gabriela.Riemekasten@uksh.de; 51 Fondazione IRCCS Policlinico San Matteo University of Pavia, 27100 Pavia- PV Italy, Germany; l.scelsi@smatteo.pv.it; 52 Clinic for General and Interventional Cardiology/Angiology, Herz- und Diabeteszentrum NRW, Ruhr-Universität Bochum, 32545 Bad Oeynhausen, Germany; wscholtz@hdz-nrw.de; 53 Dept. Cardiology and Angiology, Faculty of Medicine, Slovak Medical University and National Institute of Cardiovascular Diseases, 83348 Bratislava, Slovakia; simkova.iveta@gmail.com; 54 Universitätsklinikum Bonn, Medizinische Klinik und Poliklinik II, Innere Medizin-Kardiologie/Pneumologie, 53127 Bonn, Germany; Dirk.Skowasch@ukb.uni-bonn.de; 55 Pauls Stradins Clinical University Hospital, 1002 Riga, Latvia; andris.skride@gmail.com; 56 Klinik Löwenstein, Medizinische Klinik I, 74245 Löwenstein, Germany; gerd.staehler@klinik-loewenstein.de; 57 Universitäts-Herzzentrum Freiburg - Bad Krozingen, Klinik für Angeborene Herzfehler und Pädiatrische Kardiologie, 79189 Freiburg, Germany; brigitte.stiller@uniklinik-freiburg.de; 58 2nd Critical Care Department, Attikon University Hospital, National and Kapodistrian University of Athens, 12462 Athens, Greece; itsagkaris@med.uoa.gr; 59 Pulmonary Hypertension Center, Dept. Clnical, Anestesiologic and Cardiovascular Sciences, University of Rome La Sapienza, 00185 Rome, Italy; dario.vizza@uniroma1.it; 60 VU Medical Center Amsterdam, 1081 Amsterdam, The Netherlands; a.vonk@vumc.nl; 61 Universitätsklinikum des Saarlandes, Innere Medizin V, 66421 Homburg, Germany; Heinrike.Wilkens@uniklinikum-saarland.de; 62 Universitätsklinikum Leipzig, Medizinische Klinik und Poliklinik I, Abteilung für Pneumologie, 04103 Leipzig, Germany; hubert.wirtz@medizin.uni-leipzig.de; 63 Thoraxklinik Heidelberg gGmbH, Zentrum für Pulmonale Hypertonie, 69126 Heidelberg, Germany; ekkehard.gruenig@med.uni-heidelberg.de; 64 Universitätsklinik Köln- Herzzentrum, Klinik III für Innere Medizin, 50937 Köln, Germany; stephan.rosenkranz@uk-koeln.de

**Keywords:** congenital heart disease, pulmonary hypertension, pulmonary arterial hypertension, adults, observational, survival, targeted therapy

## Abstract

Introduction: Pulmonary hypertension (PH) is a common complication in patients with congenital heart disease (CHD), aggravating the natural, post-operative, or post-interventional course of the underlying anomaly. The various CHDs differ substantially in characteristics, functionality, and clinical outcomes among each other and compared with other diseases with pulmonary hypertension. Objective: To describe current management strategies and outcomes for adults with PH in relation to different types of CHD based on real-world data. Methods and results: COMPERA (Comparative, Prospective Registry of Newly Initiated Therapies for Pulmonary Hypertension) is a prospective, international PH registry comprising, at the time of data analysis, >8200 patients with various forms of PH. Here, we analyzed a subgroup of 680 patients with PH due to CHD, who were included between 2007 and 2018 in 49 specialized centers for PH and/or CHD located in 11 European countries. At enrollment, the patients’ median age was 44 years (67% female), and patients had either pre-tricuspid shunts, post-tricuspid shunts, complex CHD, congenital left heart or aortic disease, or miscellaneous other types of CHD. Upon inclusion, targeted therapies for pulmonary arterial hypertension (PAH) included endothelin receptor antagonists, PDE-5 inhibitors, prostacyclin analogues, and soluble guanylate cyclase stimulators. Eighty patients with Eisenmenger syndrome were treatment-naïve. While at inclusion the primary PAH treatment for the cohort was monotherapy (70% of patients), with 30% of the patients on combination therapy, after a median observation time of 45.3 months, the number of patients on combination therapy had increased significantly, to 50%. The use of oral anticoagulants or antiplatelets was dependent on the underlying diagnosis or comorbidities. In the entire COMPERA-CHD cohort, after follow-up and receiving targeted PAH therapy (*n* = 511), 91 patients died over the course of a 5-year follow up. The 5-year Kaplan–Meier survival estimate for CHD associated PH was significantly better than that for idiopathic PAH (76% vs. 54%; *p* < 0.001). Within the CHD associated PH group, survival estimates differed particularly depending on the underlying diagnosis and treatment status. Conclusions: In COMPERA-CHD, the overall survival of patients with CHD associated PH was dependent on the underlying diagnosis and treatment status, but was significantly better as than that for idiopathic PAH. Nevertheless, overall survival of patients with PAH due to CHD was still markedly reduced compared with survival of patients with other types of CHD, despite an increasing number of patients on PAH-targeted combination therapy.

## 1. Introduction

Pulmonary hypertension (PH) is one of the most severe complications of congenital anomalies of the heart and/or the great vessels (CHD) [1]. The presence of PH aggravates the natural, post-operative or post-interventional course of the underlying anomaly and impacts disease burden and outcome. The various types of CHD (i.e., pre-/post-tricuspid shunts, complex lesions) differ substantially in disease, clinical manifestation, functionality, and clinical outcomes, and the prevalence, characteristics, and impact of PH largely depend on the nature of the anomaly. However, scientific data on the impact of PH on the various forms of CHD remain sparse.

According to current guidelines [2], PH is generally defined as a mean pulmonary artery pressure (mPAP) ≥25 mmHg, although an even lower threshold of 20 mmHg has recently been proposed by the Sixth World Symposium on Pulmonary Hypertension [3]. Pre-capillary PH, characterized by a pulmonary artery wedge pressure (PAWP) ≤15 mmHg and a pulmonary vascular resistance (PVR) >3 Wood units (WU), is distinguished from postcapillary PH, where PAWP is ≥15 mmHg, and PVR may be <3 WU (isolated post-capillary) or ≥3 WU (combined post- and pre-capillary) [2,3]. The given hemodynamic definitions also apply to PH associated with CHD [2].

The clinical classification of PH comprises five major groups: pulmonary arterial hypertension (PAH, Group I); PH due to left heart disease (LHD, Group II); PH due to lung disease (Group III); chronic thromboembolic PH (Group IV); and unclear or multifactorial PH (Group V) [4]. According to guideline recommendations, PH associated with CHD is assigned to either Group I (PAH), Group II (due to LHD), or Group V (unclear/multifactorial mechanism) [1,2,5,6,7].

The incidence of PH associated with CHD has been declining in developed countries but remains prevalent despite advances in modern medicine. This is also true for Eisenmenger syndrome [6]. The prevalence of PAH ranges from 4.2% to 28% in CHD patients, and Eisenmenger syndrome develops in 3.5% to 7.1% of patients with CHD and PAH [8,9]. Women with CHD have a 33% higher risk of developing PAH than men [10], which is consistent with the female predominance in idiopathic PAH [11].

Due to the improved survival chances of both patients who have undergone successful repair of a left-right shunt and complex patients with palliative Fontan circulation who may develop non-Eisenmenger types of PH, clinical deterioration due to pulmonary vascular disease has become a common complication. In the future, this complication is likely to increase, largely determining morbidity and mortality of affected patients [6,12,13,14,15,16]. Beyond that, PH is also an evolving phenomenon in aging patients with congenital anomalies of the left heart, e.g., in aortic coarctation, congenital aortic valve disease, or congenital mitral valve disease [1].

Although numerous drugs have been approved for the treatment of PAH, the evidence for their efficacy and safety in CHD remains limited, as these patients have either been excluded from major trials or were underrepresented and inadequately characterized. Furthermore, reliable data on the outcomes of patients with PAH in CHD is comparatively limited compared with data on patients with idiopathic PAH.

The aim of the current study was to depict demographics, characteristics, treatment patterns, and outcome of patients with PH due to CHD who were included into COMPERA (Comparative, Prospective Registry of Newly Initiated Therapies for Pulmonary Hypertension), a European-based PH registry that enrolls patients with all forms of PH, and constitutes one of the largest series of patients with any form of PH or pulmonary vascular disease [17,18,19].

## 2. Patients and Methods

### 2.1. Setting

The COMPERA registry was launched in July 2007 and continues to enroll patients [20]. Since June 2009, COMPERA includes patients with all forms of PH on any pulmonary vasoactive therapy. From June 1st, 2007 until December 1st, 2018, a total of 8200 adult PH patients have been registered in COMPERA.

The registry was approved by the institutional review boards (Technical University Dresden, Germany: Master approval EK 129052007 as of 22 May 2007) of all contributing centers and written informed consent was obtained from all participating patients before the start of documentation. Guidelines on good pharmacoepidemiological practice (GPP) and data protection guidelines were followed. As of December 1st, 2018, 49 PAH centers from 11 European countries (Austria, Belgium, Germany, Hungary, Italy, Latvia, Lithuania, Slovakia, Switzerland, the Netherlands, and the UK) had entered their data in COMPERA-CHD.

### 2.2. Patient Selection

Included in the current study were adults (age ≥18 years) with any form of PAH in CHD on targeted PAH-drug treatment for the PAH, either as mono- or combination therapy. In addition, the registry allowed the documentation of patients with Eisenmenger physiology not receiving targeted PAH treatment.

The PAH diagnosis was established by the participating investigators from expert centers according to current guidelines [3,21], but was not adjudicated by a third party. Eisenmenger syndrome was defined as PH associated with CHD stemming from an initially large, non-restrictive intra- or extracardiac communication with systemic-to-pulmonary shunt, that induced progressive pulmonary vascular disease, shunt reversal, and central cyanosis.

Also included and designated as “Non-Eisenmenger-PAH-treated” were CHD patients who had undergone previous corrective surgery or interventions for an intra- or extracardiac systemic-to-pulmonary shunt, with PAH associated with prevalent systemic-to-pulmonary shunts, or PAH with small/coincidental defects.

Moreover, patients with univentricular circulation after cavo-pulmonary anastomosis (modified Fontan operation) were documented if they received targeted PAH medication for “pulmonary vascular disease or dysfunction”.

Targeted PAH medication included endothelin receptor antagonists (ERA), phosphodiesterase type-5 inhibitors (PDE5i), riociguat (as the only licensed soluble guanylate cyclase (sGC) stimulator), or prostanoids (including selexipag as an oral prostacyclin receptor agonist).

### 2.3. Data Documentation

Patients were included on a consecutive basis with data collected prospectively at the time of diagnosis (baseline), and usually in six-month intervals or whenever the patient had a predefined clinical event (death, transplantation, PH-related hospitalization, deterioration in World Health Organization (WHO) functional class (WHO-FC), any unscheduled change in PAH therapy, or other serious adverse events).

Documentation included demographics (age, sex), type of PH according to the 2013 nice classification, WHO-FC, six-minute walking distance (6MWD), selected laboratory variables, hemodynamic data obtained by previous cardiac catheterization, arterial oxygen saturation, and detailed information about the PAH medication [3].

For the documentation of adults with PAH due to CHD, the treatment centers were offered either a standard internet-based documentation form or an extended documentation form for specialist centers capturing additional information (i.e., type and repair status of cardiac malformation and additional hemodynamic parameters). The collected, comprehensive data allowed the description and categorization of subgroups of CHD in greater detail and with higher reliability than in previous reports.

Out-of-range data or missing values were automatically queried during data entry or in the context of statistical plausibility checks at regular intervals. The validity of the entered data was verified by comparison of registry data with source data in the patient files in the context of independent on-site monitoring in approximately 70% of the participating centers.

In order to compare the findings on PAH in CHD patients with those in other PAH populations, incident patients (inclusion within six months of diagnosis) with idiopathic PAH—according to European Society of Cardiology (ESC) and the European Respiratory Society (ERS) guidelines—who were enrolled into COMPERA after January 1st, 2009, were selected for comparison.

For CHD patients, time since inclusion into COMPERA was evaluated, for idiopathic PAH time since diagnosis. Survival analyses were restricted to an observation time of 5 years.

### 2.4. Statistical Analysis

Descriptive statistics in terms of percentages, median with interquartile range (IQR), or mean with standard deviations were provided to describe characteristics of the study population. Comparison of categorical variables was conducted by the χ^2^-test or the Fisher’s exact test. Group differences of continuous variables were tested with a t-test if normal distribution was assumed, or the Mann–Whitney test otherwise. A *p*-value < 0.05 was considered significant. Survival was compared using Kaplan–Meier analysis, and *p*-values of the Breslow test are shown. Tests were performed without adjustment for multiple testing. IBM SPSS Statistics 24.0 (IBM, Armonk, NY, US) was used for all statistical analyses.

The author(s) of this manuscript have certified that they comply with the Principles of Ethical Publishing in the American Journal of Cardiology [22].

## 3. Results

### 3.1. Patient Demographics and Baseline Characteristics

Out of 8200 adult PH patients enrolled in COMPERA at the time of data analysis, 680 (8.3%) had a diagnosis of PAH due to CHD, and were therefore eligible for the present analysis.

Patient demographics and baseline characteristics are summarized in Table 1, Table 2 and Table 3. All patients had undergone a complete non-invasive evaluation upon enrollment. Cardiac catheterization had been performed in 487 patients (71.6%). In patients without catheterization, the PAH diagnosis was established through non-invasive imaging and clinical evaluation.

In the entire cohort of 680 patients with CHD-associated PAH, 320 (47.1%) had a proven Eisenmenger syndrome, 167 (24.6%) had “non-Eisenmenger PAH”, and 7 (1.0%) had a Fontan circulation. Another 186 (27.4%) patients with CHD were on a targeted PAH medication but could not be clearly categorized on the basis of the data entered.

**Figure 1:**
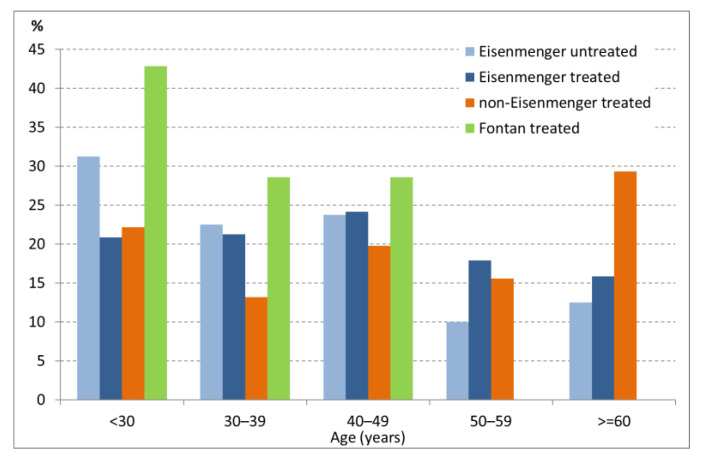
Age distribution of the population with CHD-associated pulmonary arterial hypertension (PAH). Data represent the percentage of patients from each subgroup in the respective age groups. CHD, congenital heart disease.

The median age was 44 years (range 18–87 years), and 66.6% (*n* = 453) were female. More than half of the patients were in the 3rd, 4th, or 5th decade of life (*n* = 379, 55.7%); 148 patients were younger than 30 years (21.8%); and 153 patients (22.5%) were in the 6th decade of life or older (22.5%) (Figure 1).

At first assessment, 26.6% (*n* = 181), 57.6% (*n* = 392), and 4.0% (*n* = 27) of the patients were in WHO-FC I/II, III, and IV, respectively. WHO-FC was not documented in 80 patients (11.8%). At the time of inclusion, the mean 6MWD (assessed in 454 patients) was 367 ± 120 m.

### 3.2. Type of Congenital Heart Defect

The underlying main diagnoses of CHD were sub-classified into five groups (Table 4): pre-tricuspid shunts (*n* = 213); post-tricuspid shunts (*n* = 325); complex forms of CHD (*n* = 121); left-sided heart disease, congenital aortic valve anomalies and obstruction of the aorta (*n* = 9); and “other CHD”, a group of 12 patients with diagnoses of pulmonary artery stenosis (*n* = 3), AV valve anomalies (*n* = 2), and other entities (*n* = 5), as well as two patients for whom the type of CHD was not reported in detail.

The most common underlying CHD was ventricular septal defect (29.3%), followed by atrial septal defect (27.4%), atrioventricular septal defect (11.6%), patent ductus arteriosus Botalli (5.9%), and pulmonary atresia with ventricular septal defect (4.4%).

A total of 167 patients (63.3%) had developed PAH after previous reparative cardiac surgery for CHD (operated; *n* = 264). For 55 of these patients (20.8%), the dates of PAH diagnosis and/or surgery were not available.

Down syndrome was present in 92 patients and most of them had a complete atrioventricular-septal defect (AVSD) (58.7%) or a ventricular septal defect (27.2%) as underlying CHD.

### 3.3. Medication and Treatment Strategy

**Figure 2:**
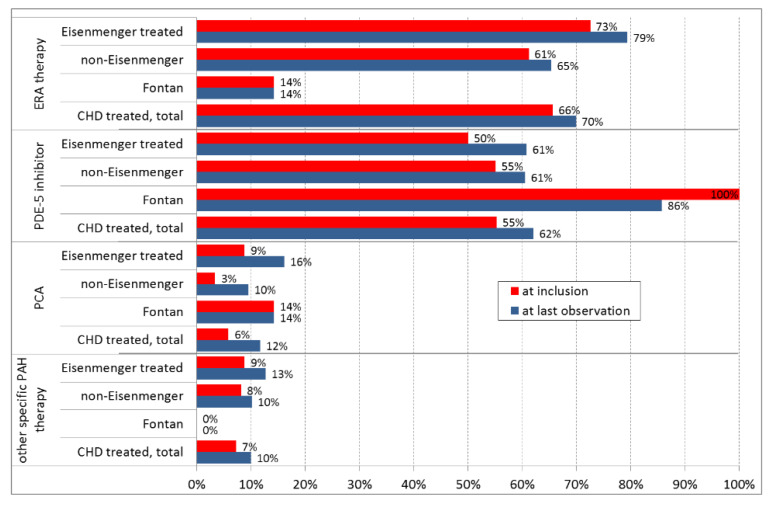
Drug classes for targeted PAH therapy in the patients with CHD-associated PAH at inclusion and at last observation (mean/median observation time of 50.5/45.3 months respectively; for 512 patients with at least one follow-up). “Other therapy” includes soluble guanylate cyclase (sGC) stimulator, tyrosine kinase inhibitor, calcium channel blockers, and other PAH-specific trial therapies. Abbreviations: PCA, prostanoids.

At baseline, of the entire CHD-associated PAH group of 680 patients, 600 (88.2%) received targeted PAH therapy. Of those 600 patients, 389 (65%) received endothelin receptor antagonists; 353 (59%) phosphodiesterase type-5 inhibitors; 35 (5.8%) prostanoids; 17 (2.8%) a soluble guanylate cyclase stimulator; a single patient a tyrosine kinase inhibitor; and 24 (4.0%) received calcium channel blockers (Figure 2).

Phosphodiesterase type-5 inhibitors were the preferred drug class in the small Fontan cohort (*n* = 7; 7/7 of these patients received this treatment).

Regarding baseline use within each drug class, among endothelin receptor antagonists, bosentan (*n* = 276) was more often used than ambrisentan (*n* = 49), sitaxentan (*n* = 26), or macitentan (*n* = 38), while among phosphodiesterase type-5 inhibitors (*n* = 353), sildenafil (*n* = 286) was more often used than tadalafil (*n* = 67). With regard to sitaxentan, it should be noted that the registry was established at a time when this drug had not yet been withdrawn from the market.

Considering changes in targeted PAH medications over time, the primary treatment choice was maintained in the majority of patients. Changes in drug prescription within the different drug classes of targeted PAH therapy between inclusion and at last observation (median 45.3 months) are depicted in Figure 2 for 512 patients with at least one follow-up.

Among all treated patient groups (*n* = 600), the primary treatment strategy at inclusion was monotherapy in the majority of patients. In the Eisenmenger and non-Eisenmenger-PAH patients, 33% and 27%, respectively, were on initial combination therapy with at least two PAH drugs. In the small Fontan group (*n* = 7), all but one patient were on monotherapy.

**Figure 3:**
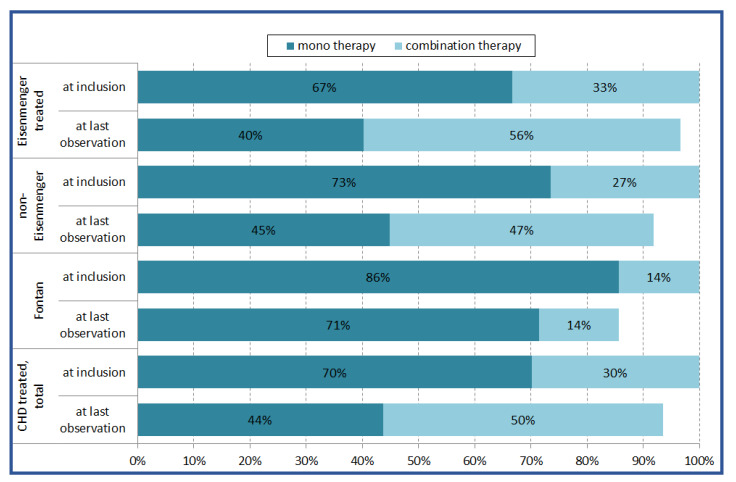
Targeted mono- or combination therapy in 512 CHD patients with PAH, with at least one follow-up using different drug classes at inclusion and at last observation (mean/median observation time of 50.5/45.3 months respectively). CHD, congenital heart disease; PAH, pulmonary arterial hypertension

During the observation period, the treatment pattern changed over time, as significantly more patients were on combination therapy at their last observation (Figure 3).

**Figure 4:**
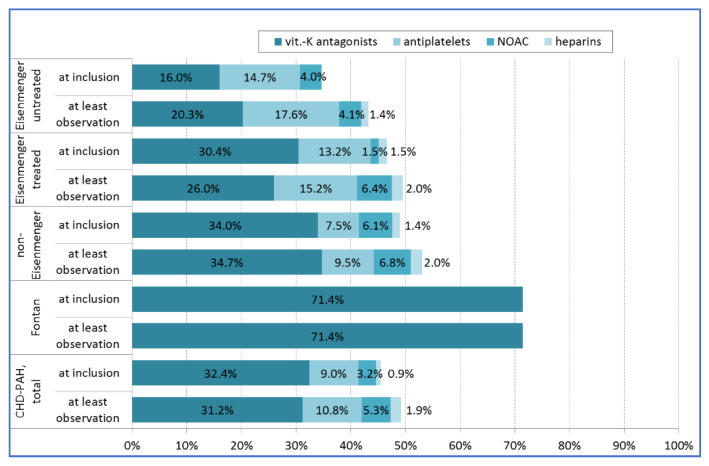
Anticoagulation/platelet inhibition regime in patients with CHD-associated PAH at inclusion and at last observation. Abbreviations: CHD, congenital heart disease; NOAC, non-vitamin K antagonists; vit, vitamin; PAH, pulmonary arterial hypertension

The use of anticoagulants or platelet inhibitors varied among groups, and according to the time of observation. The use of anticoagulants or platelet inhibitors was lowest in untreated Eisenmenger patients and highest in patients with Fontan circulation (Figure 4).

### 3.4. Survival

The survival status of patients receiving PAH medication was evaluated in 511 patients of the CHD cohort and 1326 patients from the idiopathic PAH cohort. Five-year survival could be ascertained in 78% (400 out of 511) of the CHD-cohort and 66% (872 out of 1326) of the idiopathic PAH cohort. The intermediate loss to follow-up was 3.9% (20 out of 511) in the CHD group, and 2.6% (35 out of 1326) in the idiopathic PAH group.

**Figure 5:**
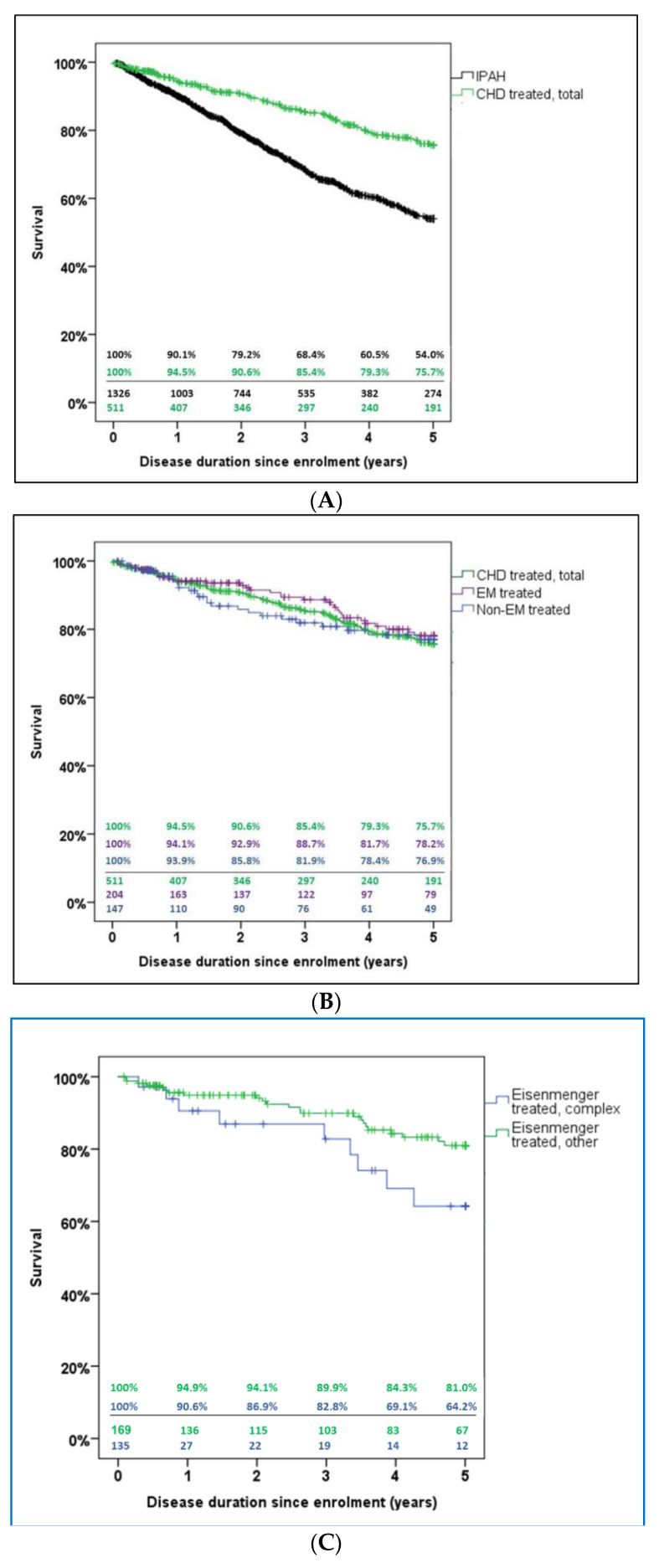
Kaplan–Meier 5-year survival estimates. (**A**) Comparison of all patients who underwent targeted PAH treatment for either congenital heart disease-associated pulmonary hypertension (CHD treated, total) or incident idiopathic PAH. The 5-year Kaplan–Meier survival estimate was 76% for PAH due to CHD and 54% for idiopathic PAH (*p* < 0.001). (**B**) Comparison of subgroups of patients with CHD and PAH under targeted PAH medication: (1) CHD-treated, total (green); (2) Eisenmenger-patients-treated (EM treated; purple); (3) non-Eisenmenger-PAH-patients (Non-EM treated; blue). The overall 5-year survival estimate was 76%, compared with 78% for the Eisenmenger patients, and 77% for the non-Eisenmenger-PAH-patients (*p* = 0.384). (**C**) Comparison of subgroups of patients with Eisenmenger syndrome due to complex or simple CHD. The 5-year Kaplan–Meier survival estimate was 81% for Eisenmenger syndrome caused by simple CHD and 64% for Eisenmenger syndrome due to complex CHD (*p* = 0.063). Kaplan–Meier survival estimates (top) and number of cases still under observation (bottom) are shown in tabular form. Abbreviations: CHD, congenital heart disease; non-EM, non-Eisenmenger-PAH-patients; PAH, pulmonary arterial hypertension.

In patients receiving targeted PAH therapy at enrollment, 91 CHD patients and 419 idiopathic PAH patients died during the 5-year follow-up period. Survival of CHD patients with targeted PAH treatment was superior to that of idiopathic PAH patients; the 5-year (Kaplan–Meier) survival estimate was 76% for CHD with PAH and 54% for idiopathic PAH (*p* < 0.001) (Figure 5A).

Within the entire CHD cohort that received targeted PAH therapy at enrollment, the 5-year Kaplan–Meier survival estimate was 76% overall, 78% for Eisenmenger patients, and 77% for non-Eisenmenger-PAH patients (*p* = 0.384) (Figure 5B). Thus, the non-Eisenmenger-PAH patients, had 5-year survival rates comparable to those of the Eisenmenger patients.

The current data show that the survival rate in Eisenmenger patients is also affected by the complexity of the CHD. The survival rate of 121 patients with Eisenmenger syndrome due to a complex CHD (including transposition of the great arteries, congenitally corrected transposition of the great arteries, double-inlet ventricle, double-outlet right ventricle—Fallot type, double-outlet right ventricle with transposition of the great arteries, Ebstein´s anomaly, tetralogy of Fallot, tricuspid atresia, pulmonary atresia with intact ventricular septum, pulmonary atresia with ventricular septal defect, and truncus arteriosus), was worse than the survival rate of patients in whom simple CHD had caused an Eisenmenger syndrome. Among the Eisenmenger patients receiving targeted PAH therapy at enrollment, nine patients in the complex-CHD and 23 patients in the simple-CHD cohort died during the 5-year follow-up period. Survival of patients with Eisenmenger syndrome due to a simple CHD was superior to survival of those with Eisenmenger syndrome caused by a complex CHD; the 5-year Kaplan–Meier survival estimate was 81% for simple CHD and 64% for complex CHD (*p* = 0.063) (Figure 5C).

## 4. Discussion

The international COMPERA-CHD registry provides information on the characteristics, treatment patterns, and long-term outcomes in a large number of patients with PAH caused by CHD, in the modern treatment era. This is of paramount importance as there are relatively limited data on targeted PAH therapy and outcomes in adults with PAH resulting from CHD compared with idiopathic PAH [1,23,24,25,26,27]. Furthermore, the existing data are mostly derived from small, uncontrolled studies with few patients, predominantly with simple congenital heart defects, and short monitoring periods [28].

Evidence for the efficacy, safety, and tolerability of PAH therapies (endothelin receptor antagonists, phosphodiesterase type-5 inhibitors/soluble guanylate cyclase stimulators, prostanoids) is available from numerous randomized clinical trials of acquired PAH, demonstrating improvements in exercise tolerance, quality of life, and reduction of morbidity and mortality events in PAH [29,30,31,32]. However, such data may be ambiguous or even misleading for the management of PAH in CHD.

Even if patients with CHD and PAH were included in the majority of trials, neither the type of CHD nor the CHD-specific hemodynamic status have been sufficiently considered, and data have not, for the most part, been presented separately from data on non-congenital forms of PAH.

Accordingly, there is a shortage of evidence about targeted PAH therapies for CHD with PAH, and it remains unclear whether data from current studies on other forms of PAH can be applied to PAH patients with CHD [23,33].

As long as data from randomized controlled trials are not available for PAH from CHD, data from registries, which include larger patient populations and longer observation periods than clinical trials, are valuable to characterize PAH associated with CHD in terms of demographics, clinical presentation, risk factors, and outcomes, as well as PAH-targeted therapies or supportive measures. However, similar to controlled clinical trials, most PAH registries have so far also failed to differentiate between CHD subtypes, and have merged the characteristics, treatments, and outcomes of different patient groups, thus precluding the analysis of details on CHD subgroups [12,13,34,35,36,37,38].

Accordingly, the special situation of CHD patients is not yet adequately addressed in the current PAH guidelines. The German consensus-based recommendations for the management of patients with pulmonary hypertension state that the ESC/ERS guidelines on pharmacotherapy in PAH from CHD are focused on patients with Eisenmenger syndrome. The main reason for this may be the currently available evidence, but this also implies that the recommendations do not fully acknowledge the breadth, heterogeneity, and complexity of CHD patients with associated PAH [1]. The COMPERA registry provides for the first time such data in Europe on a broad scale.

The COMPERA registry is one of the largest structured, non-interventional (observational), prospective international registries for patients with any form of PH, providing a unique opportunity to obtain detailed information on epidemiological and clinical features, treatment patterns, and outcomes in a sufficient number of patients with various forms of CHD with PAH. Within this registry, we analyzed a group of 680 adults with CHD and PAH, with a focus on PAH-targeted therapy, supportive treatment, and outcome. As a unique feature, COMPERA-CHD not only included adults with CHD associated PAH who were receiving PAH-targeted therapy, but also a number of Eisenmenger patients who were not receiving any targeted therapy.

### 4.1. Demographics, Hemodynamics, and Treatment

This cohort of patients with CHD associated PAH comprised almost all types of congenital heart anomalies, including post-tricuspid shunts (*n* = 325), followed by pre-tricuspid shunts (*n* = 213), and complex anomalies (*n* = 121).

The mean age of each subgroup of patients with CHD associated PAH at the time of inclusion was significantly lower than the mean age of the included idiopathic PAH patients, but higher than the mean age in any other registry of patients with PAH in CHD [12,13,34,35,36,37].

Despite many parallels between PAH in CHD and idiopathic PAH, COMPERA revealed several attributes that, depending on the underlying congenital cardiovascular anomaly, distinguish CHD-associated PAH from idiopathic PAH. These include a more favorable hemodynamic profile due to a better maintenance of cardiac output and a more favorable right ventricular response, in CHD-associated PAH [8].

One major difference is that, compared with other forms of PAH, the cause of PAH in CHD is often multifactorial, but commonly secondary to a left-to-right shunt lesion leading to increased pulmonary artery flow and/or pressure. Other important predisposing factors for pulmonary vascular disease in CHD include previous Fontan operation and left heart obstructive disease causing post-capillary PH. A further distinction from other forms of PAH is that, in CHD, timing and extent of the development of PAH depends on various different factors, e.g., the pre- or post-tricuspid localization of the primary left-to right shunt [39]. For example, Eisenmenger syndrome will evolve even in large defects at the atrial level much later in life compared with defects at the ventricular or great artery levels.

Moreover, the data from the COMPERA registry confirm the perception that patients with PAH from CHD, unlike patients with other forms of PAH, are often clinically stable for years or decades and remain at a low WHO-FC until an advanced age.

This may explain why, despite the existence of Eisenmenger syndrome, a considerable number of patients without PAH-targeted therapy enrolled in COMPERA. Pertinent to this, patients in the untreated Eisenmenger cohort were younger and had milder clinical symptoms. In addition, in this group, the 6MWD was substantially higher than that in treated Eisenmenger patients (392 ± 118 vs. 354 ± 121 m). These observations may indicate that healthcare centers did not apply targeted PAH therapy indiscriminately but followed the current guidelines, which recommend PAH-targeted therapy depending on clinical symptoms and WHO-FC.

In contrast, however, due to the accumulating evidence for the safety and efficacy of targeted therapy, the majority of patients with CHD and PAH included in COMPERA were treated with targeted PAH medications.

Among the various drug classes, at baseline, endothelin receptor antagonists and phosphodiesterase type-5 inhibitors were by far the predominantly prescribed compounds, whereas prostanoids and soluble guanylate cyclase stimulators were only rarely used.

Initially, the majority of the 600 treated patients with CHD associated PAH were on monotherapy, although a significant number of patients was put on combination therapy (Figure 3). Furthermore, utilization of combination therapy increased over time (with the exception of Fontan patients), with approximately half of the patients being on combination therapy at the time of last observation (Figure 3).

At that point, the therapeutic approach for adults with PAH and CHD differs from the approach for those with idiopathic PAH, as there are only few data to support routine up-front/early sequential oral combination therapy for Eisenmenger syndrome [1,6,26,39,40,41,42]. In adults with PAH and CHD, sequential combination therapy is frequently initiated only upon symptomatic deterioration or if predefined, and often center-specific treatment goals are not achieved [43].

### 4.2. Co-Medication with Anticoagulants or Antiplatelets

Another important subject is the use of oral anticoagulants or antiplatelets in CHD-associated PAH, which appears to differ from that in idiopathic PAH [14]. A recent, controversially debated study from the COMPERA group revealed a survival benefit from using anticoagulation in patients with idiopathic PAH; however, for other forms of PAH, the evidence remained inconclusive [18]. In patients with Eisenmenger syndrome, the situation is even more complex, as they usually have both increased bleeding and increased thrombotic risk due to changes in platelet function, platelet counts, and coagulation parameters. So far, no data exist suggesting that oral anticoagulation has a beneficial effect on morbidity or mortality in Eisenmenger patients. Consequently, current guidelines on CHD do not recommend the routine use of oral anticoagulation for these patients [1,6,44,45].

This approach is also reflected in the COMPERA data. At baseline, most patients (up to 66%) in both subgroup with the Eisenmenger syndrome and the non-Eisenmenger-PAH subgroups were not anticoagulated using vitamin K antagonists, and only a few patients received oral non-vitamin K antagonists.

In contrast, most patients with Fontan circulation (71%) were on oral anticoagulants to prevent thromboembolic events, reflecting the common treatment strategy, at least in many adults with early modifications of the Fontan operation [46].

Surprisingly, at least 13.4% of the Eisenmenger patients were on antiplatelets, although currently no reliable data exist on a protective effect of Aspirin or Clopidogrel for this condition. The application of antiplatelets can perhaps be attributed to the unproven belief that they may have fewer negative effects on the bleeding tendency in cyanotic patients than oral anticoagulants. On the contrary, the inhibitory effect of the always diminished count of platelets in cyanotic patients can be devastating.

### 4.3. Survival

Although mortality has decreased in adults with CHD, morbidity is still high due to residua, sequelae, or complications, and many patients develop pathologies of the pulmonary vascular bed, complicating the natural history of the particular anomaly [4,23,47,48]. In adults with PAH from CHD, all-cause mortality is more than two-fold, and the rate of health service utilization is three-fold higher compared with CHD without PAH [38]. Moreover, the survival prospects of treatment-naïve patients with Eisenmenger syndrome has not improved since the 1960s, if confounding factors, such as immortal time bias, are considered [49].

The survival rates of patients with different types of PAH have been published based on a few studies and national registries, demonstrating that patients with PAH in CHD had a better survival rate than those with idiopathic PAH. The current data from the COMPERA registry confirm this, as the 5-year survival of patients with PAH from CHD was significantly better than that of idiopathic PAH-patients.

It is noteworthy that the 5-year survival rate of treated patients with PAH from CHD in COMPERA was 76%, i.e., lower than in some previous studies. However, Diller et al. recently reported a similar 5-year survival rate of 74.9% (95% CI 67.6–83.1%) for the Eisenmenger patients in the German National Registry for CHD [24]. In contrast, Manes et al. showed, in their mono-centric analysis, estimated survival rates of 87%, 86%, and 36% after follow-up for up to 20 years in 192 patients with Eisenmenger syndrome, PAH in prevalent systemico-pulmonary shunts, and PAH after repair, respectively [42].

The unfavorable survival rate in COMPERA may be attributable to the fact that the PAH in several of the enrolled patients was due to a complication of complex CHD (*n* = 121; 18%). In support of this possibility, Diller et al. reported that patients with Eisenmenger syndrome due to complex CHD had a significantly worse prognosis than patients with a simple CHD [50].

Comparing COMPERA CHD patients treated for Eisenmenger syndrome with those who had any form of PAH from CHD but no Eisenmenger syndrome, there was no significant difference between the groups in terms of 5-year survival.

Under targeted PAH medication, the survival rate of non-Eisenmenger-PAH patients appears to be less favorable than that of Eisenmenger patients, especially over a time period of 1 to 4 years.

These data are in accordance with the results from the Spanish REHAP registry [12], as well as data from Thailand [25]. Both of these studies also found that patients with Eisenmenger syndrome had a better survival rate than patients with PAH after previous defect closure [12,25].

### 4.4. Limitations

Strengths include the large sample size of prospectively enrolled patients with PAH caused by CHD, the inclusion of all types of PAH from CHD, the “real-life” setting, and the relatively long observation period.

This study has limitations, as it is a voluntary, prospective registry and not a prospective randomized clinical trial. However, COMPERA enrolls consecutive patients on a prospective basis and several control measures have been implemented to ensure high data quality, including independent on-site source data monitoring. Still, missing values were a relevant limitation of this study.

Observational data such as ours are prone to various types of bias including selection bias, incomplete outcome bias (attrition bias), immortal time bias, and survivor bias [51,52,53].

Statistical measures to reduce confounding factors, such as propensity score matching or multivariable risk-adjusted modelling, were not applied, and therefore group differences at baseline have to be considered for all results and conclusions.

COMPERA did not document in detail medications other than PAH-approved drugs and anticoagulants, and did not determine therapeutic adherence, which is considered an integral component of pharmaceutical care practice and patient healthcare.

As idiopathic PAH patients in the present series were older than patients in previous PH registries, it cannot be excluded that some patients diagnosed with idiopathic PAH had comorbidities or confounding factors that may have led to a mixed etiology of PH. However, all participating centers had expertise in the management of PH and evaluated their patients according to the current guidelines. Moreover, a similar trend regarding the age of idiopathic PAH patients has been reported from other PH registries [54,55]. Co-morbidities were only assessed at inclusion in COMPERA and their increasing presence at follow up might have affected survival.

Finally, the sample of patients may not represent the pattern of adults with PAH in the community and does not represent the typical population of CHD seen by a general practitioner or a non-CHD expert cardiologist.

## 5. Conclusions

PAH is still a serious problem for numerous patients with CHD and there are many uncertainties regarding optimal medical treatment. In the future, it is expected that an increasing number of patients will develop PAH with complex CHD or pulmonary vascular diseases in the univentricular heart after a Fontan operation. The current data confirm the still-unfavorable prognosis and reduced survival rates of patients with PAH from CHD, despite the availability of a variety of PAH-targeted drugs, application modalities (e.g., oral, inhaled, subcutaneous, intravenous) and therapeutic strategies (e.g., mono, dual, or triple therapy), and palliative interventions (e.g., percutaneous Pott´s shunt).

The estimated survival rates for patients with CHD associated PAH at 5 years was only 76%, but was nevertheless significantly better than those for idiopathic PAH patients. The best survival rate was seen in patients with Eisenmenger syndrome.

The results of this study imply that it must be ascertained in any case, that the particularities of CHD are taken into account in diagnosis and treatment, as the management of PAH-CHD is often different from other forms of PAH. Accordingly, such patients should always be managed with cooperation between CHD specialists and PAH specialists, preferably in tertiary care settings.

In the future, the registry will provide data on specific patient cohorts for PAH-CHD-targeted therapeutic approaches and allow detailed survival analysis of these patients.

## Figures and Tables

**Table 1:** Demographics and clinical characteristics at enrollment.

		IPAH (*n* = 1481)		PAH-CHD Total (*n* = 680)		ES Untreated (*n* = 80)		ES Treated (*n* = 240)		Non-ES-PAH Treated (*n* = 167)		Fontan Treated (*n* = 7)	
Age		66.3 ± 15.5		45.5 ± 16.8		40.1 ± 14.6		43.8 ± 15.1		47.6 ± 18.3		31.9 ± 9.4	
Sex	male	604	40.8%	227	33.4%	26	32.5%	76	31.7%	55	32.9%	3	42.9%
	female	877	59.2%	453	66.6%	54	67.5%	164	68.3%	112	67.1%	4	57.1%
FC	unknown	78	5.3%	80	11.8%	30	37.5%	19	7.9%	14	8.4%	4	57.1%
	I	3	0.2%	22	3.2%	1	1.3%	8	3.3%	5	3.0%	0	0%
	II	178	12.0%	159	23.4%	11	13.8%	65	27.1%	44	26.3%	3	42.9%
	III	997	67.3%	392	57.6%	36	45.0%	136	56.7%	101	60.5%	0	0%
	IV	225	15.2%	27	4.0%	2	2.5%	12	5.0%	3	1.8%	0	0%
6-min-walk distance (m)		294 ± 123		367 ± 120		392 ± 118		354 ± 121		381 ± 120		475 ± 50	

Abbreviations: CHD, congenital heart disease; ES, Eisenmenger syndrome; FC, functional class according to Perloff; IPAH, idiopathic pulmonary arterial hypertension; non-ES-PAH, patients with congenital heart disease-associated pulmonary arterial hypertension but without Eisenmenger syndrome; PAH-CHD; congenital heart disease-associated pulmonary arterial hypertension. Age and 6-min-walk distance are given in mean ± SD.

**Table 2:** Treatment characteristics of the patients with congenital heart disease-associated pulmonary arterial hypertension. “Non-Eisenmenger-PAH treated” comprises patients with congenital heart disease-associated pulmonary arterial hypertension but without Eisenmenger syndrome who received targeted PAH treatment. Abbreviations: PAH, pulmonary arterial hypertension.

Treatment Characteristics	Number of Patients Included
**CHD-PAH total**	**680**
Eisenmenger - untreated	80 (11.8%)
Eisenmenger - treated	240 (35.3%)
Non-Eisenmenger-PAH - treated	167 (24.6%)
Fontan - treated	7 (1.0%)
Not categorized	186 (27.4%)
**Targeted PAH medication**	
Endothelin receptor antagonists (ERA)	389 (57.2%)
Phosphodiesterase type-5 inhibitors (PDE5i)	353 (51.9%)
Prostanoids	35 (5.1%)
Soluble guanylate cyclase (sGC) stimulator	17 (2.5%)
Tyrosine kinase inhibitor	1 (0.1%)
**Treatment strategy and supportive treatment**	
Monotherapy with targeted PAH medication	408 (60.0%)
Combination therapy with targeted PAH medication	192 (28.3%)
Oral anticoagulation: Vitamin K antagonists	217 (31.9%)
Oral anticoagulation: Non-vitamin K antagonists (NOAC)	30 (4.4%)
Antiplatelet therapy (Aspirin, Clopidogrel)	68 (10.0%)

**Table 3:** Targeted PAH treatment of the patients with congenital heart disease-associated pulmonary arterial hypertension. “Non-Eisenmenger-PAH treated” comprises patients with congenital heart disease-associated pulmonary arterial hypertension but without Eisenmenger syndrome who received targeted PAH treatment. Abbreviations: ERA, endothelin receptor antagonists; PAH, pulmonary arterial hypertension; PDE5-I, phosphodiesterase type-5 inhibitors; sGC-stimulator, soluble guanylate cyclase (sGC) stimulator. Comparative tests were only performed between treated Eisenmenger and non-Eisenmenger patients.

Treatment Group	Number of Patients Included (Total)	Targeted PAH Medication	*n* (%)	*p*-Value	Mono-/Combination-Therapy	*p*-Value
CHD-associated PAH - total	680	ERA PDE5-I Prostanoid sGC-stimulator Tyrosine kinase inhibitor	389 (57.2%) 353 (51.9%) 35 (5.1%) 17 (2.5%) 1 (0.1%)		408 (60.0%)/ 192 (28.3%)	
Eisenmenger, treated	240	ERA PDE5-I Prostanoid sGC-stimulator Tyrosine kinase inhibitor	172 (71.7%) 134 (55.8%) 20 (8.3%) 7 (2.9%) 0		152 (63.3%)/ 88 (36.7%)	
Non-Eisenmenger-PAH, treated	167	ERA PDE5-I Prostanoid sGC-stimulator Tyrosine kinase inhibitor	100 (59.9%) 97 (58.1%) 7 (4.2%) 6 (3.6%) 1 (0.6%)	0.013 0.652 0.099 0.703 0.410	121 (72.5%)/ 46 (27.5%)	0.054
Fontan, treated	7	ERA PDE5-I Prostanoid sGC-stimulator Tyrosine kinase inhibitor	1 (14.3%) 7 (100 %) 1 (14.3%) 0 0		6 (85.7%)/ 1 (14.3%)	
Not categorized, treated	187	ERA PDE5-I Prostanoid sGC-stimulator Tyrosine kinase inhibitor	116 (62.4%) 115 (61.8%) 7 (3.8%) 4 (2.2%) 0		129 (69.4%)/ 57 (30.6%)	

**Table 4:** Subgroups of adult patients with PAH, and types of congenital heart defects.

		*n* (%)
1. Pre-tricuspid shunts (*n* = 213)	Persisting foramen ovale	5 (0.7)
	Atrial septal defect	186 (27.4)
	Partial atrioventricular septal defect	4 (0.6)
	Partial anomalous pulmonary venous return	16 (2.4)
	Total anomalous pulmonary venous return	1 (0.1)
	details not stated	1 (0.1)
2. Post-tricuspid shunts (*n* = 325)	Complete atrioventricular septal defect	79 (11.6)
	Ventricular septal defect	199 (29.3)
	Patent ductus arteriosus Botalli	40 (5.9)
	Aortopulmonary window	6 (0.9)
	details not stated	1 (0.1)
3. Complex anomalies (*n* = 121)	Complete transposition of great arteries	19 (2.8)
	Congenitally corrected transposition of great arteries	12 (1.8)
	Double-outlet right ventricle with transposition of great arteries	5 (0.7)
	Truncus arteriosus	4 (0.6)
	Tricuspid atresia	12 (1.8)
	Double-inlet ventricle	13 (1.9)
	Pulmonary atresia with intact ventricular septum	1 (0.1)
	Fallot´s Tetralogy	13 (1.9)
	Double-outlet right ventricle—Fallot type	9 (1.3)
	Pulmonary atresia with ventricular septal defect	30 (4.4)
	Ebstein’s anomaly	2 (0.3)
	details not stated	1 (0.1)
4. Left heart disease/aortic valve, and aortic anomalies (*n* = 9)	Aortic coarctation	2 (0.3)
	Aortic valve stenosis	5 (0.7)
	Subaortic stenosis	1 (0.1)
	Aortic valve regurgitation	1 (0.1)
5. Other congenital cardiac anomalies (*n* = 12)	Atrioventricular valve anomalies	2 (0.3)
	other	5 (0.7)
	Pulmonary artery stenosis	3 (0.4)
	details not stated	2 (0.3)
